# Stimulation of Hemolysis and Eryptosis by α-Mangostin through Rac1 GTPase and Oxidative Injury in Human Red Blood Cells

**DOI:** 10.3390/molecules28186495

**Published:** 2023-09-07

**Authors:** Sumiah A. Alghareeb, Jawaher Alsughayyir, Mohammad A. Alfhili

**Affiliations:** Department of Clinical Laboratory Sciences, College of Applied Medical Sciences, King Saud University, Riyadh 12372, Saudi Arabiajalsughayyir@ksu.edu.sa (J.A.)

**Keywords:** mangostin, eryptosis, hemolysis, oxidative stress, anticancer

## Abstract

Background: Chemotherapy-related anemia is prevalent in up to 75% of patients, which may arise due to hemolysis and eryptosis. Alpha-mangostin (α-MG) is a polyphenolic xanthonoid found in the mangosteen tree (*Garcinia mangostana*) whose antitumor medicinal properties are well-established. Nevertheless, the potential toxic effects of α-MG on red blood cells (RBCs) have, as of yet, not been as well studied. Methods: RBCs were exposed to 1–40 μM of α-MG for 24 h at 37 °C. Hemolysis and related markers were measured using colorimetric assays, eryptotic cells were identified through Annexin-V-FITC, Ca^2+^ was detected with Fluo4/AM, and oxidative stress was assessed through H_2_DCFDA using flow cytometry. The toxicity of α-MG was also examined in the presence of specific signal transduction inhibitors and in whole blood. Results: α-MG at 10–40 μM caused dose-dependent hemolysis with concurrent significant elevation in K^+^, Mg^2+^, and LDH leakage, but at 2.5 μM it significantly increased the osmotic resistance of cells. A significant increase was also noted in Annexin-V-binding cells, along with intracellular Ca^2+^, oxidative stress, and cell shrinkage. Moreover, acetylcholinesterase activity was significantly inhibited by α-MG, whose hemolytic potential was significantly ameliorated by the presence of BAPTA-AM, vitamin C, NSC23766, and isosmotic sucrose but not urea. In whole blood, α-MG significantly depleted intracellular hemoglobin stores and was selectively toxic to platelets and monocytes. Conclusions: α-MG possesses hemolytic and eryptotic activities mediated through Ca^2+^ signaling, Rac1 GTPase activity, and oxidative injury. Also, α-MG leads to accelerated cellular aging and specifically targets platelet and monocyte populations in a whole blood milieu.

## 1. Introduction

Alpha-mangostin (α-MG) is a polyphenolic xanthonoid isolated from the bark, dried sap, and fruit pericarp of the mangosteen tree (*Garcinia mangostana*). In Ayurveda, tree extracts have been widely used for gastrointestinal, suppurative, and ulcerative symptoms [1]. In particular, α-MG has been shown to possess a wide array of bioactive properties including anticancer, antioxidant, anti-inflammatory, and antimicrobial activities. Previous studies have demonstrated the antiproliferative effects of α-MG against liver [2], colon [3], skin [4], breast [5], prostate [6], pancreas [7], and lung [8] cancer both in vitro and in vivo.

Chemotherapy-induced anemia, observed in up to 75% of patients undergoing treatment [9], may arise due to myelosuppression and inflammatory damage leading to defective erythropoiesis. Direct red blood cell (RBC) toxicity causing hemolysis or eryptosis has also been recognized as an underlying mechanism behind anemia caused by chemotherapeutic agents [10]. Eryptosis serves to eliminate defective, aged, and infected RBCs prior to intravascular hemolysis as eryptotic cells display phosphatidylserine (PS) on their surface which serves as a binding site for phagocytes. Inordinate and premature eryptosis may, however, be instigated by a variety of stimuli including xenobiotics, heavy metal toxicity, infections, nutritional deficiencies, diabetes, liver and kidney disease, and malignancy. Moreover, eryptotic cells adhere to endothelial walls, predisposing patients to microcirculatory injuries such as thrombosis, ischemia, and hypoxia [11].

Molecular mechanisms of eryptosis include intracytoplasmic Ca^2+^ accumulation, reactive oxygen species (ROS) overload, cell membrane scrambling, disrupted channel trafficking, dehydration and cell shrinkage, and ceramide buildup. A host of signal transduction pathways have also been identified in RBCs whose role in cell death has been described. These include caspases, p38 mitogen-activated protein kinase (MAPK), AMP-activated protein kinase, casein kinase 1α (CK1α), protein kinase C (PKC), cGMP-dependent protein kinase type I, Janus kinase 3, receptor-interacting proteins 1 and 3, and mixed lineage kinase domain like pseudokinase (MLKL) [11].

Although α-MG has promising potential as an antitumor agent, its interaction with human RBCs remains largely unknown. In this work, we aim to investigate the toxicity of α-MG on RBCs and identify associated mechanisms.

## 2. Results

### 2.1. α-MG Induces Dose-Dependence Hemolysis

**Figure 1:**
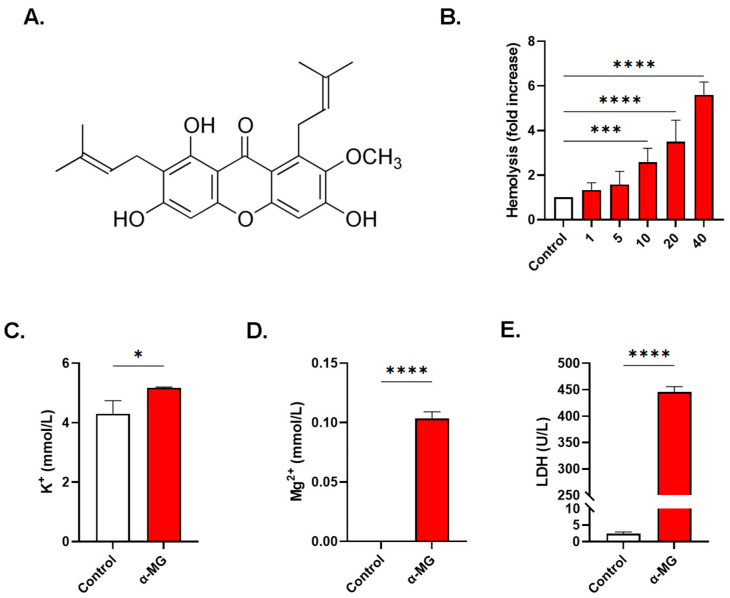
α-MG induces hemolysis. (**A**) Chemical structure of α-MG. (**B**) Dose-responsive hemolytic activity of α-MG (fold change). Leakage of (**C**) K^+^, (**D**) Mg^2+^, and (**E**) LDH into the supernatant. Results are shown as means ± SEM (*n* = 9). * (*p* < 0.05), *** (*p* < 0.001), and **** (*p* < 0.0001).

As shown in Figure 1B, compared to negative control values of 0.96 ± 0.17 folds, incubation of cells with α-MG caused dose-responsive hemolysis, attaining statistical significance at 10 µM (2.53 ± 0.51 folds, *p* < 0.0001), 20 µM (3.22 ± 0.88 folds, *p* < 0.0001), and 40 µM of α-MG (5.61 ± 0.58 folds, *p* < 0.0001).

Canonical markers of hemolysis were also significantly elevated in cells treated with 40 µM of α-MG including K^+^ (4.30 ± 0.44 vs. 5.17 ± 0.02 mmol/L, *p* < 0.05, Figure 1C), Mg^2+^ which increased from undetectable levels to 0.10 ± 0.01 mmol/L (*p* < 0.0001, Figure 1D), and LDH (2.45 ± 0.51 vs. 445.40 ± 10.30 U/L, *p* < 0.0001, Figure 1E).

### 2.2. α-MG Improves the Osmotic Resistance of RBCs

**Figure 2:**
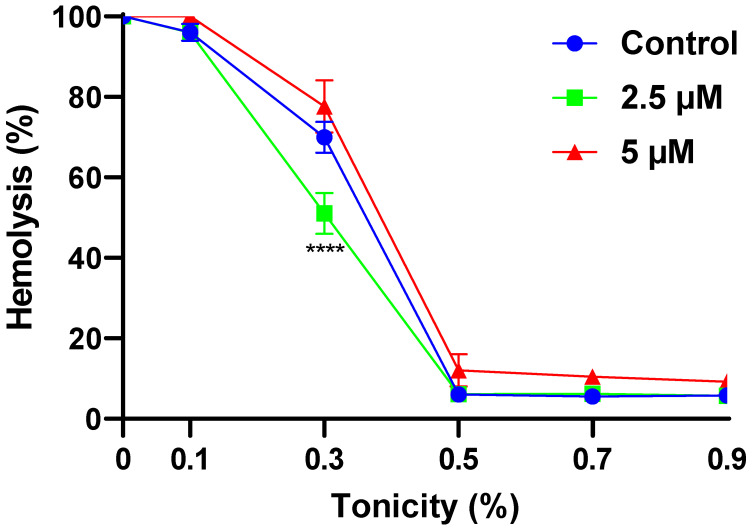
Effect of α-MG on hypotonic hemolysis. Results are shown as means ± SEM (*n* = 9). **** (*p* < 0.0001).

Figure 2 demonstrates that the treatment of cells with 2.5 µM of α-MG significantly inhibits hypotonic hemolysis at 0.3% tonicity compared to the negative control (69.94 ± 6.67% vs. 51.10 ± 8.81%, *p* < 0.0001).

### 2.3. α-MG Stimulates Eryptosis

**Figure 3:**
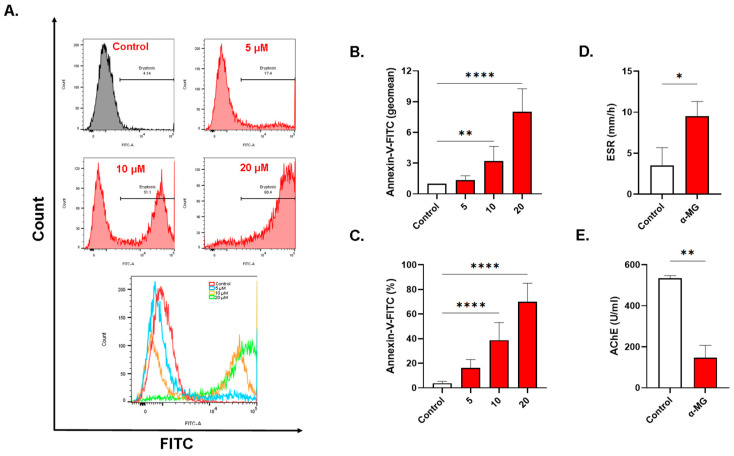
α-MG causes premature aging of RBCs. (**A**) Representative histograms of Annexin-V-FITC fluorescence. (**B**) Geomean of Annexin-V-FITC fluorescence (fold change). (**C**) Percentage of eryptotic cells. (**D**) ESR. (**E**) AChE activity. Results are shown as means ± SEM (*n* = 9). * (*p* < 0.05), ** (*p* < 0.01), and **** (*p* < 0.0001).

As seen in Figure 3B, α-MG significantly increased the geomean of Annexin-V-FITC fluorescence to 3.19 ± 1.44 folds (10 µM, *p* = 0.0044) and 8.02 ± 2.25 folds (20 µM, *p* < 0.0001). The percentage of PS-exposing cells also increased from the negative control values of 3.75 ± 1.60% to 16.26 ± 6.83% (5 µM, *p* = 0.0764), 38.80 ± 14.30% (10 µM, *p* < 0.0001), and 69.93 ± 15.13% (20 µM, *p* < 0.0001) as shown in Figure 3C. The ESR was significantly elevated in exposed cells in comparison to the negative control values (3.50 ± 0.49 to 9.50 ± 0.44, *p* = 0.0213, Figure 3D) while AChE activity was significantly diminished from the negative control values of 534.4 ± 9.0 U/mL to 146.6 ± 49.5 U/mL (*p* < 0.01, Figure 3E).

### 2.4. α-MG Causes Cell Shrinkage and Surface Granularity

**Figure 4:**
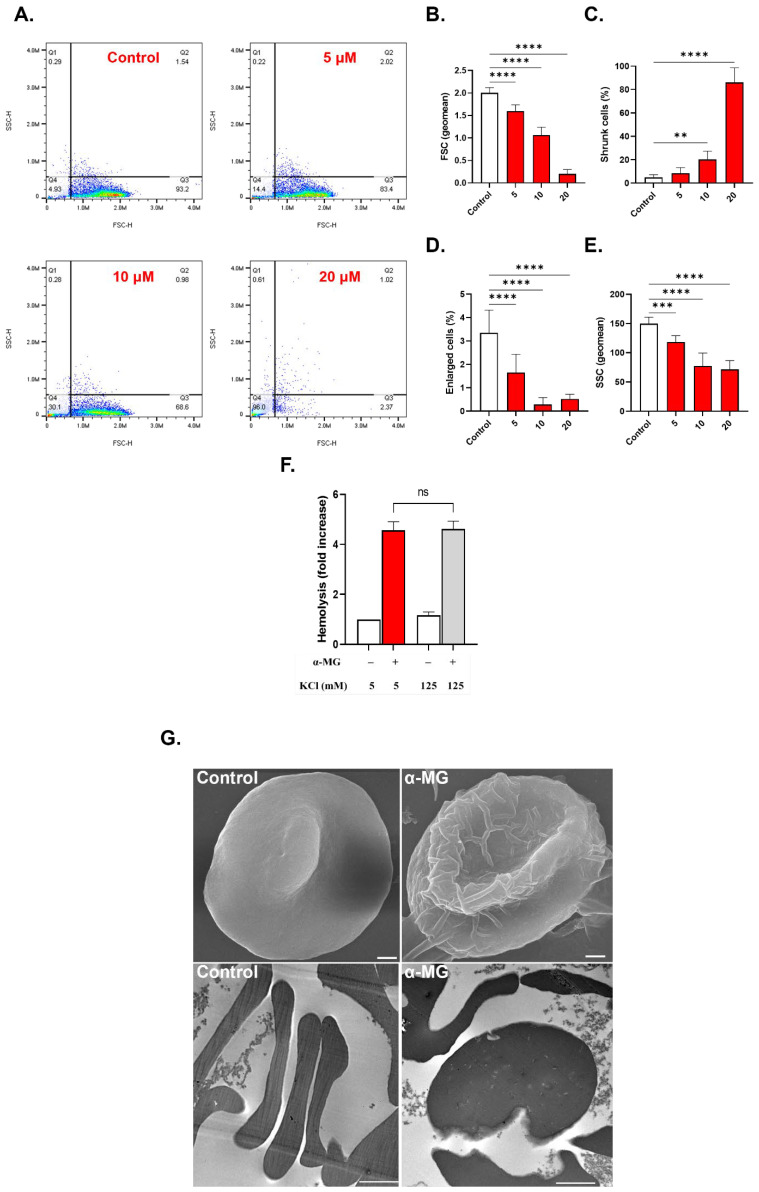
Effect of α-MG on RBC morphology. (**A**) Representative dot plots of SSC-H and FSC-H distribution of cells. (**B**) Geomean of FSC in arbitrary units (a.u.). (**C**) Percentage of shrunk cells. (**D**) Percentage of enlarged cells. (**E**) Geomean of SSC in a.u. (**F**) Effect of 125 mM extracellular KCl on hemolysis. (**G**) Electron micrographs of cells (SEM X7,000; TEM X15,000). Scale bar: 1 μm (SEM) and 2 μm (TEM). ns indicates no statistical significance, while ** (*p* < 0.01), *** (*p* < 0.001), and **** (*p* < 0.0001).

Cells treated with 5, 10, and 20 µM of α-MG exhibited significant changes in FSC (1.59 ± 0.14 a.u. (*p* < 0.0001), 1.06 ± 0.18 a.u. (*p* < 0.0001), and 0.20 ± 0.1 a.u. (*p* < 0.0001), respectively) compared to negative control values of 2.0 ± 0.12 a.u (Figure 4B). The percentage of shrinking cells significantly increased from 5.0 ± 2.23% in the case of negative control to 20.38 ± 7.0% (*p* = 0.0038) and 86.08 ± 12.48% (*p* < 0.0001) after treatment with 10 µM and 20 µM of α-MG, respectively (Figure 4C). Furthermore, as shown in Figure 4D, substantially fewer enlarged cells were detected at 5 µM (1.65 ± 0.79%, *p* < 0.0001), 10 µM (0.35 ± 0.21%, *p* < 0.0001), and 20 µM of α-MG (0.54 ± 0.22%, *p* < 0.0001) in comparison to negative control values of 3.34 ± 0.9793%.

Also, Figure 4E shows that SSC values of 149.6 ± 11.24 a.u. in negative control cells were significantly decreased to 118.2 ± 11.07 a.u. (*p* < 0.0001), 77.63 ± 21.98 a.u. (*p* < 0.0001), and 71.67 ± 14.85 a.u. (*p* < 0.0001) in cells treated with 5, 10, and 20 µM of α-MG, respectively. Notably, increasing extracellular KCl to 125 mM failed to prevent α-MG-induced hemolysis (4.57 ± 0.33 folds vs. 4.61 ± 0.31 folds, *p* = 0.9978, Figure 4F). Degmacyte formation was also noted upon electron microscopy examination (Figure 4G).

### 2.5. α-MG Elevates Cytosolic Calcium

**Figure 5:**
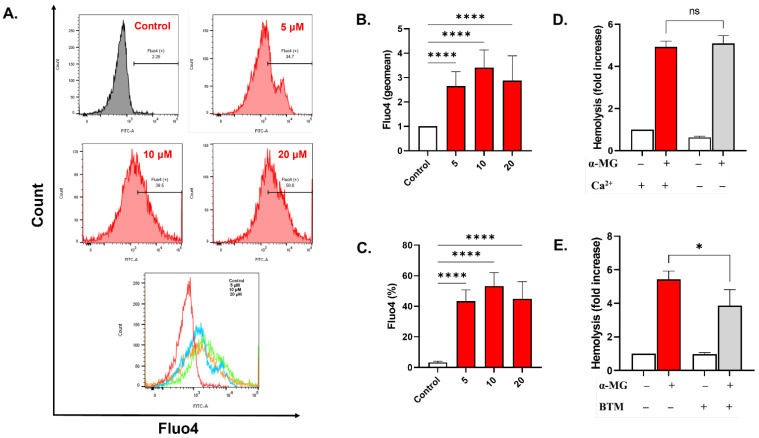
α-MG raises cytosolic Ca^2+^ levels. (**A**) Representative histograms of Fluo4 fluorescence. (**B**) Geomean of Fluo4 fluorescence (fold change). (**C**) Percentage of cells with Ca^2+^ accumulation. (**D**) Effect of Ca^2+^ elimination on hemolysis. (**E**) Inhibition of hemolysis by 10 μM of BTM. Results are shown as means ± SEM (*n* = 9). ns indicates no statistical significance, while * (*p* < 0.05) and **** (*p* < 0.0001).

As shown in Figure 5B, 5, 10, and 20 µM of α-MG induced a significant increase in the geomean of Fluo4 fluorescence to 2.652 ± 0.59 folds (5 µM, *p* < 0.0001), 3.40 ± 0.73 folds (10 µM, *p* < 0.0001), and 2.40 ± 0.98 folds (20 µM, *p* < 0.0001). The percentage of cells with high Ca^2+^ was 43.37 ± 7.38% (*p* < 0.0001), 53.25 ± 8.78% (*p* < 0.0001), and 44.92 ± 11.22% (*p* < 0.0001), respectively, relative to the negative control which was 3.244 ± 0.86% (Figure 5C). Although hemolysis was not prevented by the elimination of extracellular Ca^2+^ (Figure 5D), it was nonetheless significantly ameliorated by the addition of BTM (5.42 ± 0.28 folds vs. 3.85 ± 0.55 folds, *p* = 0.019, Figure 5E).

### 2.6. α-MG Promotes Oxidative Stress

**Figure 6:**
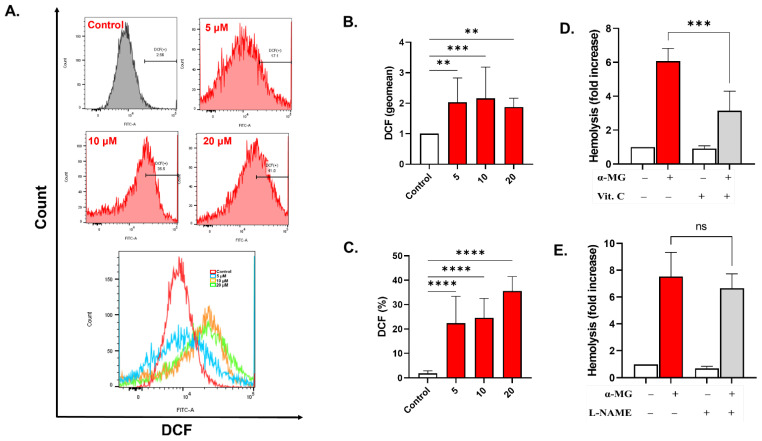
α-MG elicits oxidative damage. (**A**) Representative histograms of Fluo4 fluorescence. (**B**) Geomean of Fluo4 fluorescence (fold change). (**C**) Percentage of cells with Ca^2+^ accumulation. (**D**) Inhibition of hemolysis by 1 mM of vitamin C. (**E**) Effect of 20 μM of L-NAME on hemolysis. Results are shown as means ± SEM (*n* = 9). ns indicates no statistical significance, while ** (*p* < 0.01), *** (*p* < 0.001), and **** (*p* < 0.0001).

The geomean of DCF fluorescence, shown in Figure 6B, significantly increased to 2.03 ± 0.8 folds (5 µM, *p* = 0.0028), 2.158 ± 01.03 folds (10 µM, *p* = 0.0008), and 1.87 ± 0.29 folds (20 µM, *p* = 0.004). The percentage of oxidized cells (Figure 6C) in negative control samples (1.87 ± 1.05%) was also elevated after exposure to 5, 10, and 20 µM of α-MG, increasing to 22.36 ± 11.03% (*p* < 0.0001), 24.62 ± 7.90% (*p* < 0.0001), and 35.56 ± 5.924% (*p* < 0.0001), respectively. Congruently, vitamin C (Figure 6D) but not L-NAME (Figure 6E) significantly inhibited the hemolytic activity of α-MG (6.06 ± 0.37 vs. 3.15 ± 0.57 folds *p* < 0.0001).

### 2.7. Rac1 GTPase Is Essential for the Hemolytic Activity of α-MG

**Figure 7:**
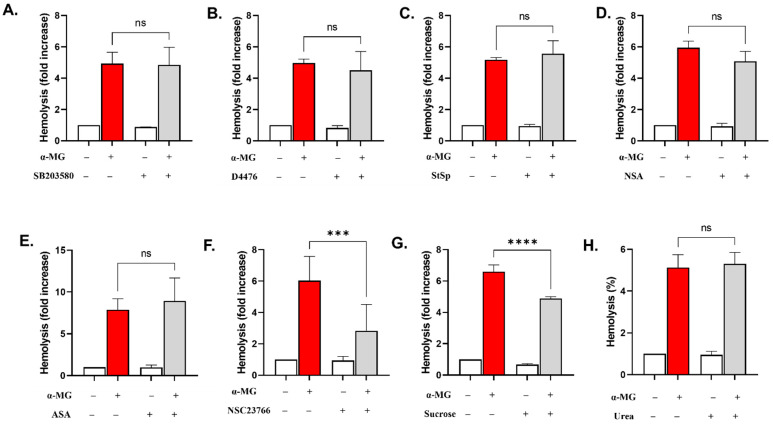
α-MG-induced hemolysis is ameliorated by NSC23766 and isosmotic sucrose. Effect of (**A**) SB (100 µM), (**B**) D4476 (20 µM), (**C**) StSp (1 µM), (**D**) NSA (500 nM), (**E**) ASA (25 µM), (**F**) NSC23766 (100 µM), (**G**) sucrose (250 mM), and (**H**) urea (300 mM) on hemolysis. Results are shown as means ± SEM (*n* = 9). ns indicates no statistical significance, while *** (*p* < 0.001) and **** (*p* < 0.0001).

No statistically significant decrease in the hemolytic rate of α-MG was observed in the presence of SB203580 (Figure 7A), D4476 (Figure 7B), StSp (Figure 7C), NSA (Figure 7D), or ASA (Figure 7E). However, in cells cotreated with NSC23766, a significant reduction in hemolysis was observed from 6.04 ± 1.53 folds to 2.84 ± 1.66 folds (*p* < 0.001), as revealed in Figure 7F. This was also the case in the presence of sucrose (6.59 ± 1.27 folds to 4.88 ± 0.35 folds, *p* < 0.0001, Figure 7G).

### 2.8. α-MG Elicits Distinct Alterations in Whole Blood

**Figure 8:**
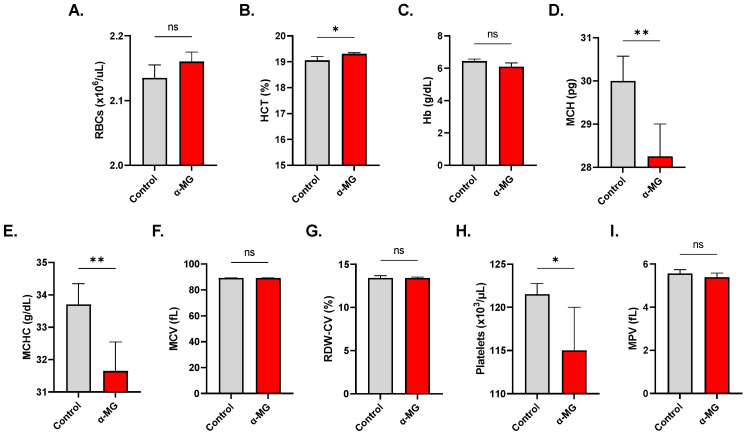
Depletion of corpuscular Hb and antiplatelet activity of α-MG. (**A**) RBC count. (**B**) HCT. (**C**) Hb. (**D**) MCH. (**E**) MCHC. (**F**) MCV. (**G**) RDW-CV. (**H**) Platelet count. (**I**) MPV. Results are shown as means ± SEM (*n* = 9). ns indicates no statistical significance, while * (*p* < 0.05) and ** (*p* < 0.01).

A slight but significant increase in the HCT from 19.0 ± 0.21% to 19.27 ± 0.15% (*p* < 0.05) was observed following treatment with 40 µM of α-MG (Figure 8B). MCH (29.93 ± 0.69 vs. 28.17 ± 1.06 pg, *p* < 0.01) and MCHC (33.57 ± 0.82 vs. 31.58 ± 1.23 g/dL, *p* < 0.01) were significantly reduced in treated whole blood as shown in Figure 8E,H, respectively. Furthermore, Figure 8H reveals that α-MG was selectively toxic to platelets whose numbers significantly decreased to 115.8 ± 4.76 × 10^3^/µL from control values of 121.20 ± 2.93 × 10^3^/µL (*p* < 0.05).

**Figure 9:**
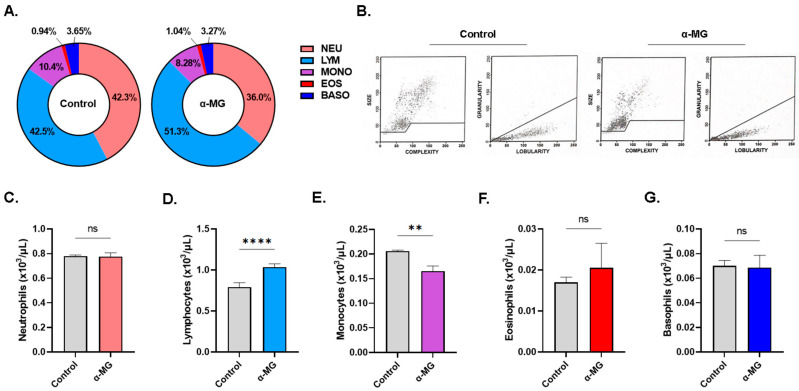
Effect of α-MG on white blood cell subsets. (**A**) Differential count of leukocytes. (**B**) Representative dot plots of size, complexity, and lobularity of leukocytes. Viability of neutrophils (**C**), lymphocytes (**D**), monocytes (**E**), eosinophils (**F**), and basophils (**G**). Results are shown as means ± SEM (*n* = 6). ns indicates no statistical significance, while ** (*p* < 0.01) and **** (*p* < 0.0001).

It was also noted that α-MG caused significant disruption in leukocyte proportions (Figure 9A) as neutrophil percentage significantly decreased from 42.30 ± 0.64% to 36.0 ± 2.32% (*p* < 0.05), lymphocyte percentage significantly increased from 42.50 ± 0.81% to 51.30 ± 2.44% (*p* < 0.01), and monocyte percentage significantly decreased from 10.40 ± 0.42% to 8.28 ± 0.34% (*p* < 0.01). These changes were accompanied by cell shrinkage and increased lobularity as seen in Figure 9B. Accordingly, a significant increase in lymphocyte count (0.80 ± 0.051 vs. 1.03 ± 0.07 × 10^3^/µL, *p* < 0.0001) and a decrease in monocyte count (0.20 ± 0.02 vs. 0.17 ± 0.01 × 10^3^/µL, *p* < 0.01) were also evident in Figure 9D,E, respectively.

## 3. Materials and Methods

### 3.1. Chemicals and Reagents

All chemicals were of the highest purity and were purchased from Solarbio Life Science (Beijing, China) unless otherwise noted. A stock solution of α-MG (CAS #6147-11-1) was prepared by dissolving 5 mg in 1.21 mL of dimethylsulfoxide (DMSO) and stored at −80 °C. Phosphate-buffered saline (PBS) contained 137 mM NaCl, 2.7 mM KCl, 10 mM Na_2_HPO_4_, and 1.8 mM KH_2_PO_4_, pH 7.4, while Ringer solution was composed of 125 mM NaCl, 5 mM KCl, 1 mM MgSO_4_, 32 mM HEPES, 5 mM glucose, and 1 mM CaCl_2_, pH 7.4. KCl-Ringer was prepared by replacing NaCl and KCl with 125 mM KCl, while Sucrose-Ringer was prepared by replacing NaCl with 250 mM sucrose. Urea was added to standard Ringer solution at 300 mM [12].

### 3.2. Ethical Approval and Blood Collection

This study was approved by the Ethical Committee of King Saud University (E-23-7764). Blood was collected from 10 healthy volunteers in lithium heparin and EDTA vacutainer tubes, and RBCs were isolated by centrifugation at 2500 RPM for 15 min at room temperature. Following repeated washing in PBS and removal of the upper 10% of the sediment, cells were finally suspended in Ringer solution at 1:3 *v*/*v* and stored at 4 °C for a maximum of 24 h. The purity of the RBC suspension was validated using the BC-6200 hematology analyzer (Mindray Medical International Limited, Shenzhen, China). Exposure to α-MG (1–40 μM) was performed in Ringer solution at a hematocrit of 5% at 37 °C for 24 h.

### 3.3. Hemolysis

Control and treated cells were sedimented by centrifugation (13,000 RPM for 1 min) and the absorbance of the supernatant was measured at 405 nm using the LMPR-A14 microplate reader (Labtron Equipment Ltd., Surrey, UK). A positive control (i.e., 100% hemolysis), prepared by suspending the cells in ddH_2_O, was run in parallel and percent hemolysis was expressed as a fold change relative to negative control values [13].

### 3.4. Potassium Leakage

The K^+^ content of extracellular space was measured using the Blood Potassium Content Assay Kit (Solarbio). In brief, cells were treated with the vehicle (0.1% DMSO) or 40 µM of α-MG at 37 °C for 24 h and the supernatant was assayed for K^+^ leakage. Sodium tetraphenylboron in the reaction mixture reacts with K^+^ to form potassium tetraphenylboron which is insoluble in water. The resultant turbidity (λ_max_ = 520 nm) is proportional to the concentration of K^+^ in the sample.

### 3.5. Magnesium Release

Release of intracellular Mg^2+^ into the supernatant was measured using Solarbio’s Blood Magnesium Content Assay Kit. Under alkaline conditions, Mg^2+^ combines with hydroxides and turns orange–red upon reacting with the triazene dye, titan yellow (λ_max_ = 540 nm).

### 3.6. Lactate Dehydrogenase (LDH) Activity

LDH activity was assayed using Solarbio’s LDH Activity Assay Kit. In a coupled reaction, LDH converts NAD^+^ and lactic acid to NADH and pyruvate, which further reacts with 2,4 dinitrophenylhydrazine to form pyruvate dinitrobenzene. This has a brown–red color in alkaline conditions proportional in intensity to pyruvate content (λ_max_ = 450 nm). One unit of enzyme activity is defined as the amount of enzyme that catalyzes the production of 1 nM of pyruvate per minute for each mL of supernatant.

### 3.7. Osmotic Fragility

Cells were added to solutions of NaCl ranging from 0 mM to 160 mM (0–0.9% NaCl) corresponding to 0–320 mOsm with or without 2.5 µM or 5 µM of α-MG, and incubated at 37 °C for 1 h before hemolysis was measured.

### 3.8. Membrane Scrambling

Cells were stained with 1% Annexin-V-FITC for 10 min at RT away from light, and fluorescence (10,000 events) was then measured at Ex/Em of 488/512 nm with the Northern Lights flow cytometer (Cytek Biosciences, Fremont, CA, USA) [14].

### 3.9. Cellular Morphology

Forward scatter (FSC) and side scatter (SSC) were determined from 10,000 events by flow cytometry. To prepare cells for electron microscopy, the negative control and the treated samples (20 μM) were fixed in 2.5% glutaraldehyde, washed in PBS, stained with 1% osmium tetraoxide, washed again in PBS, and finally dried in 50–100% ethanol. Samples were coated with carbon and visualized using both the JSM-7610F ultra-high resolution Schottky field emission scanning electron microscope and the JEM-1400 transmission electron microscope at 15.0 kV and 100 kV, respectively (JEOL Co., Ltd., Akishima, Tokyo, Japan) [15].

### 3.10. Acetylcholine Esterase (AChE) Activity

The enzymatic activity of AChE was measured using Solarbio’s AChE Activity Assay Kit. Briefly, AChE in negative control and experimental lysates generates thiocholine from acetylthiocholine, which reacts with 2-nitrobenzoic acid to form 5-mercaptonitrobenzoic acid whose absorbance at 412 nm is directly proportional to AChE activity. One unit of enzyme activity is the amount of enzyme that catalyzes the generation of 1 nM of 5-mercaptonitrobenzoic acid per minute for each mL of hemolysate [16].

### 3.11. Intracellular Ca^2+^

Negative control and treated cells were labeled with 2 µM of Ca^2+^ probe Fluo4/AM for 30 min at 37 °C in the dark, then washed twice in PBS (5000 RPM for 1 min) to remove excess dye. The stain was excited at 488 nm and emitted light was detected at 520 nm by flow cytometry. A total of 10,000 events were recorded. [17].

### 3.12. Oxidative Stress

General ROS indicator 2′,7′-dichlorodihydrofluorescein diacetate (Ex/Em = 488/533 nm) was incubated with the negative control and the treated cells for 30 min at 37 °C in the dark, washed twice in PBS (5000 RPM for 1 min) to remove excess dye, and the green light was quantified in 10,000 events by flow cytometry [18].

### 3.13. Erythrocyte Sedimentation Rate (ESR)

The sedimentation rate (mm/h) of RBCs in whole blood was recorded in Westergren tubes as previously reported [19].

### 3.14. Complete Blood Count (CBC)

Whole blood collected in EDTA was diluted 1:2 in PBS with and without 40 µM of α-MG, and a CBC was performed after 24 h of incubation at 37 °C using a BC-6200 hematology analyzer as previously reported.

### 3.15. Signal Transduction Analysis

Cells were either treated with the vehicle or with 40 µM of α-MG in the presence or absence of Ca^2+^ chelator BAPTA-AM (BTM; 10 µM); p38 inhibitor SB203580 (100 µM); CK1α inhibitor D4476 (20 µM); PKC inhibitor staurosporin (StSp; 1 µM); MLKL inhibitor necrosulfonamide (NSA; 0.5 µM); vitamin C (1 mM); nitric oxide synthase (NOS) inhibitor L-NAME (20 µM); cyclooxygenase inhibitor acetylsalicylic acid (ASA; 25 µM); or Rac GTPase inhibitor NSC23766 (100 µM). Hemolysis was then assessed following incubation at 37 °C for 24 h.

### 3.16. Statistical Analysis

Results are shown as means ± SEM (*n* = 9). GraphPad 9.0 (GraphPad Software, Inc., San Diego, CA, USA) was used for statistical analysis. Two groups were analyzed by Student’s *t*-test while three or more groups were analyzed by one-way ANOVA. A *p* value of <0.05 was considered statistically significant.

## 4. Discussion

α-MG is among the most extensively researched agents for chemoprevention, showing antiproliferative, proapoptotic, antiangiogenic, and antimetastatic properties against a broad spectrum of cancer cell types through a variety of mechanisms. Also, α-MG synergizes with various chemotherapeutic drugs to further enhance their apoptotic effect, which makes it an important therapeutic option for the treatment of cancer. This work unveils novel evidence of the in vitro toxicity of α-MG to human erythrocytes, which warrants cautious consideration of the compound for anticancer therapy.

Our results demonstrate that α-MG has strong hemolytic potential with profound K^+^, Mg^2+^, and LDH leakage (Figure 1). This indicates that α-MG causes physical damage in the cell membrane, which is expected to be exasperated in vulnerable patients including those with cancer, diabetes mellitus, or hemoglobinopathies [10]. A deleterious consequence of intravascular hemolysis is the release of hemoglobin which exerts oxidative damage to vascular walls, predisposing to atherosclerosis and thrombosis, along with systemic sequelae including hepatic, pancreatic, and renal insufficiency [12]. Moreover, degradation of naked hemoglobin contributes to inflammation and dysregulated immune function as a result of heme production, the turnover of which generates hemin that is known to stimulate hemolysis and eryptosis [17].

We also noted that α-MG exhibits a biphasic effect on the fragility of RBCs. At relatively low concentrations (<5 μM), the osmotic resistance of the cells seems to be increased, suggesting membrane expansion which allows the cell to accommodate more water influx before rupture ensues (Figure 2). Many compounds have been reported to intercalate in the lipid bilayer [20,21,22] and α-MG may very well pack into membrane pockets, thereby increasing cellular volume. It may also indicate that α-MG exposure leads to morphological alterations conducive of hypotonic resistance. Notably, the membrane-protective activity of α-MG against H_2_O_2_-induced oxidative hemolysis has been observed by Buravlev et al. [23] at 1 and 10 μM, which also implicates the xanthonoid in activating antioxidant defense mechanisms as evidenced by reduced thiobarbituric acid reactive substances. Regardless of the underlying mechanism, α-MG in this regard resembles the antihemolytic effects of quercetin [24] and *Ginkgo biloba* leaf extract [25].

This work also presents the pro-eryptotic effects of α-MG for the first time (Figure 3A–C), which is parallel to the apoptotic activity of α-MG reported in other cells [26,27]. Several lines of evidence have established the contribution of augmented eryptosis in a wide variety of conditions including diabetes mellitus, hyperlipidemia, hypertension, and cancer [10,28,29], among others. When RBCs lose the asymmetrical arrangements of phospholipid species, PS moieties are translocated to the outer membrane leaflet to serve as binding sites for stabilin-2, tim-4, or opsonins on macrophages [30]. Although the clearance of eryptotic cells prevents their eventual hemolysis, it may also reduce the number of circulating RBCs, which leads to anemia if the bone marrow fails to adequately upregulate erythropoiesis. Of note, eryptotic cells increase the risk for thrombosis and ischemia, because dead RBCs adhere to the endothelium via transmembrane CXC chemokine ligand 16, and lose their deformability and elasticity due to increased membrane rigidity [31]. Indeed, our results show that higher ESR is observed in treated cells (Figure 3D) which reflects increased clumping and Rouleaux aggregation.

Another important marker of cellular aging is AChE activity. Inhibition of the enzyme, as induced by α-MG (Figure 3E), is typically encountered following organophosphate poisoning, but alterations in RBC morphology, as seen in anemic states, may also influence AChE activity since AChE is anchored to the RBC membrane [32]. Indeed, the current study reveals severe cellular deformation following α-MG treatment (Figure 4). Moreover, increased availability of acetylcholine to RBCs has been shown to be associated with increased cellular acidity and a weaker affinity of hemoglobin to oxygen [33]. Of note, α-MG depleted intracellular Hb stores (Figure 8D,E), further exacerbating gas exchange. AChE activity is also negatively correlated with G6PD [32], suggesting a role for the enzyme in redox balance. Although the exact function of AChE in RBCs remains uncertain, it appears very likely that it is involved in oxygen delivery and oxidative metabolism, both of which become compromised upon enzyme inhibition.

The observed shrinkage and loss of surface complexity (Figure 4) indicates cellular dehydration as a result of water efflux. The decreased cellular volume occurs due to the buildup of Ca^2+^ in the cytosol (Figure 5A–C), which activates Ca^2+^-responsive K^+^ channels leading to KCl (Figure 1C) and water loss [34]. Fragmentation upon cell death would seem to facilitate phagocytic engulfment and could carry signaling mediators to neighboring cells [35]. In fact, calpain 1, a cytoskeleton-degrading enzyme, is under the regulation of Ca^2+^ activity, and may thus account for the detected cell shrinkage (Figure 4). Likewise, many of the membrane-stabilizing enzymes, including flippases, floppases, and scramblases, are Ca^2+^-dependent, and as such, become dysregulated in response to increased Ca^2+^ activity [11], resulting in PS externalization (Figure 3A–C). Importantly, the hemolytic potential of α-MG was significantly, but not completely, abrogated by cotreatment with BTM (Figure 5E) suggesting the involvement of Ca^2+^ in driving hemolysis, similar to numerous hemolytic and eryptotic inducers such as allicin and β-lapachone. Thus, other mechanisms are indeed required for the full hemolytic activity of α-MG.

Although α-MG is reported to exert antioxidant and anti-inflammatory effects [36,37], our results demonstrate that it can also promote ROS accumulation (Figure 6A–C) as previously seen in other cell types [8,38]. Oxidative stress primes RBCs for eryptosis, especially given the vulnerability of these cells to oxygen damage. In this regard, α-MG resembles other pro-eryptotic compounds such as sanguinarine and bioymifi. It has been shown that increased ROS leads to hyperactive cation channels and Ca^2+^-dependent eryptosis [39]. Another mechanism by which ROS damage RBCs involves the formation of protein carbonyls and lipid peroxides [40]. Notably, our results also show that vitamin C can partially alleviate RBC toxicity (Figure 6D), implicating ROS as indispensable to α-MG-induced hemolysis.

The role of Rac GTPases is well established in maintaining the hexagonal organization of the cytoskeleton in healthy RBCs [41]. Previous reports [14,42] have also demonstrated that Rac GTPase is crucial for the formation of intracellular ROS by activating NADPH oxidases and NOS whose blockade did not protect RBCs from hemolysis (Figure 6E). However, inhibiting the activity of Rac GTPase with NSC23766 significantly inhibited α-MG-mediated hemolysis (Figure 7F), indicating that α-MG targets mediators upstream of NOS directed by Rac GTPase. It is important to mention that Rac is itself activated by Ca^2+^ ions and PKC, which was not essential for the hemolytic activity of α-MG (Figure 7C). Thus, α-MG could activate a Ca^2+^-Rac GTPase-ROS molecular axis, especially given its role in cytoskeletal regulation.

A number of mechanisms could explain the antihemolytic properties of sucrose against α-MG (Figure 7G) in a similar fashion to sanguinarine and geraniin. Sucrose may prevent colloid osmotic swelling by inhibiting water influx, restricting chloride efflux, or accepting hydrogen ions from α-MG. In any case, the interaction between the two chemicals is not chiefly ionic since sucrose did not aggravate the hemolytic activity of α-MG, but further research is indeed warranted.

Exposure to α-MG in a whole blood context revealed augmented selective cytotoxicity toward platelets (Figure 8H) and monocytes (Figure 9E). In congruence with these findings, Liu et al. [43] have reported that α-MG-induced platelet death is associated with reduced aggregation and morphological alterations, and required extracellular Ca^2+^. The selective cytotoxicity of α-MG can be attributed to its potential to alter the actin cytoskeletal structures and stiffness, as has been reported previously in various cell types [44]. Also, as recently demonstrated, α-MG inhibits M1 polarization of monocytes [45], but further elucidation of the mechanisms governing the toxicity of α-MG to monocytes is required. Equally important is the validation of these findings based on translational evidence from clinical trials.

## 5. Conclusions

**Figure 10:**
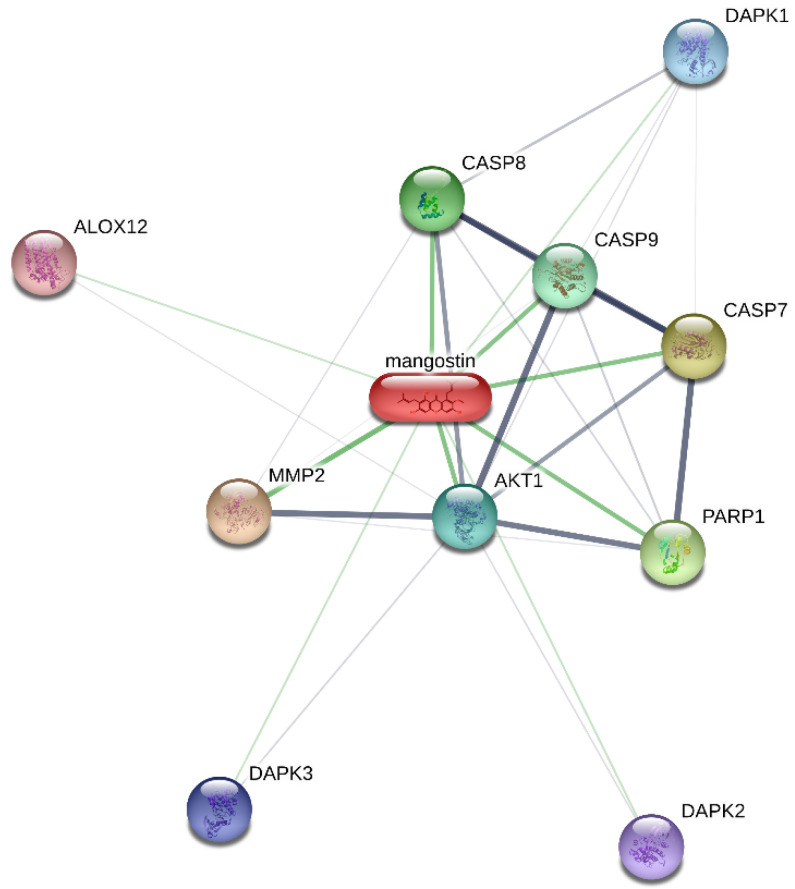
Association network of α-MG [46]. DAPK1, death-associated protein kinase 1; CASP8, caspase 8; ALOX12, arachidonate 12-lipoxygenase; CASP9, caspase 9; CASP7, caspase 7; MMP2, matrix metallopeptidase 2; AKT1, v-akt murine thymoma viral oncogene homolog 1; PARP1, poly (ADP-ribose) polymerase 1; DAPK3, death-associated protein kinase 3; DAPK2, death-associated protein kinase 2.

In conclusion, this work presents novel insights into the in vitro cytotoxic effects of α-MG in erythrocytes. Given that anemia is a common side effect of many anticancer drugs, hematological evaluation of investigational therapeutics such as α-MG is essential for safety assessment and drug development. Our study thus contributes to the current understanding of the potential therapeutic utility of α-MG. Further research in animal models and clinical trials should be directed towards elucidating the detailed mechanisms (Figure 10) through which α-MG disturbs the red cell redox balance in addition to its effects on other blood cells, and the potential protective role of known eryptotic inhibitors.

## Data Availability

The data that support the findings of this study are available on request from the corresponding author, M.A.A., upon reasonable request.
